# Knowledge of and Intention to Participate in Physical Activity Programs and Their Associated Sociodemographic Factors in People with High Blood Pressure in a Rural Area of Bangladesh: Initial Investigation from a Cluster Randomized Controlled Trial

**DOI:** 10.3390/ijerph18189561

**Published:** 2021-09-10

**Authors:** Fakir M. Amirul Islam, Mohammad Arzan Hosen, Mohammad Ariful Islam, Elisabeth A. Lambert, Bruce R. Thompson, Gavin W. Lambert, Ralph Maddison

**Affiliations:** 1 School of Health Sciences, Swinburne University of Technology, Melbourne, VIC 3122, Australia; elisabethlambert@swin.edu.au (E.A.L.); brthompson@swin.edu.au (B.R.T.); glambert@swin.edu.au (G.W.L.); 2 Organization for Rural Community Development (ORCD), Dariapur, Narail 7500, Bangladesh; arzan.hosen@orcdbd.org (M.A.H.); arifislam9120@gmail.com (M.A.I.); 3 Iverson Health Innovation Research Institute, Swinburne University of Technology, Melbourne, VIC 3122, Australia; 4 Institute for Physical Activity and Nutrition (IPAN), Faculty of Health, Deakin University, Melbourne, VIC 3125, Australia; ralph.maddison@deakin.edu.au

**Keywords:** rural area, Bangladesh, high blood pressure, Rasch analysis

## Abstract

This initial investigation aimed to investigate the knowledge of the health benefits of physical activity (PA) and attitudes towards participation in PA. The study recruited 307 people aged 30–75 years with hypertension as part of a cluster randomized controlled trial from a rural area in Bangladesh. Of the 307 participants, 135 participated less than 2.5 h of physical activity per week, from which we collected data on attitudes toward PA. Regression analysis and Rasch analysis were used. More than 85% of homemakers, employees or businesspersons were willing to take part in PA. Based on the combined score from the knowledge and attitude items, 46% of people endorsed PA programs; proportions were higher in men than women (53% vs. 41%). After adjusting for covariates, men (odds ratio, 95% confidence interval (CI) 3.50, 1.72–7.11) compared to women and people with at least primary levels of schooling (OR 3.06, 95% CI, 1.27–7.38) compared with those with no education were more likely to organize or take part in any PA programs. People have positive attitudes towards PA but do not feel obligated to participate in PA programs. Future programs are needed to promote awareness and motivational interventions for PA, especially targeting women and people with low education levels, should be developed and implemented.

## 1. Introduction

The burden of high blood pressure is considerable, with an estimated 1.13 billion people globally having hypertension, with prevalence increasing in low–middle-income countries [1]. Importantly, hypertension alone accounted for 10.8 million deaths, equivalent to 19.2% of all deaths in 2019 and 9.3% of disability-adjusted life years lost [2]. Physical inactivity and sedentary behavior are important modifiable risk factors associated with hypertension and all-cause mortality [3,4,5,6,7], with more than 5 million people dying due to being physically inactive [8,9]. Physical activity lowers the risk for all-cause mortality and chronic diseases, including type 2 diabetes, hypertension, and coronary heart disease [10,11,12].

While studies suggest that developments of various community-based public health intervention programs are needed to increase awareness in maintaining recommended healthy lifestyles, including physical activity [5,11,12,13,14,15], there is evidence to indicate that various intervention programs implemented in primary care and community settings have had limited success, with only minor or short-term changes in physical activity [16,17,18]. The key to improving an individual’s physical activity behaviors is reliant on understanding their perception, thoughts, and attitudes [19]. A previous study reported that lifestyle changes might depend on awareness and knowledge to increase motivation to adopt healthy lifestyles to manage diseases [20]. Understanding the benefits of physical activity is expected to change people’s attitudes towards physical activity, which, in turn, leads to adoption of changes to daily life [21,22].

There are data to indicate that individuals, particularly in low–middle-income countries, are generally unaware of the health benefits of physical activity [23,24]. A cross-sectional study of 450 adults among the general population in India reported that 25% of participants were unaware of the health benefit of physical activity in preventing or mitigating the detrimental effects of chronic diseases [23]. Another study of 150 people aged 20 years or older with known hypertension reported that 65% of participants did not know whether physical activity was important in controlling high blood pressure [24]. Only 26% of people had a positive attitude towards exercise programs and knowledge of and attitudes towards physical activity were higher among people with higher education, younger people, and in those with higher socioeconomic status (SES).

People generally overestimate their physical activity levels, and those who overestimate are more likely to believe that physical activity is less beneficial and express less intention to increase physical activity than those who are realistic about their activity [25,26]. A study of 453 adults in the United Kingdom [26] showed that 57% of participants self-reported that they were physically active, but half did not meet physical activity guidelines, assessed with an objective measure. Factors that contributed to the over-estimation of physical activity levels were the use of self-report measures of physical activity and higher intention to participate in physical activity. In low–middle-income countries, the prevalence of self-reported physical activity has been reported to be high, i.e., 86% in India [23], 97% in Nepal [27], 94% in Uganda [28], and 90% in Mozambique [29]. In Bangladesh, physical activity levels have ranged from 58 to 83% [30,31,32,33]. Islam [33] reported that 83% of people were physically active as measured by metabolic equivalent task-minute per week using the Global Physical Activity Questionnaire version 2 (GPAQ-2) [34]. Importantly, there seems to be a gap in the knowledge linking physical activity to better health outcomes. In India, for instance, 89% of people considered that they led a healthy lifestyle, and 83% of participants did not feel the need to increase physical activity levels. However, only a quarter of participants were aware of any health benefits of physical activity in curbing chronic disease [23]. Maintaining physical activity and limiting sedentary time are important determinants of health [6]. In individuals with high blood pressure, their knowledge, attitude, and lifestyle behavior are significant factors in controlling their blood pressure [35]. Importantly, physical activity behaviors require good knowledge of the health benefits of maintaining an active lifestyle, and positive attitudes towards adopting healthy lifestyles in order to maximize benefits [36].

Bangladesh is a low–middle-income country with a large population confronting a significant increase in chronic diseases, including hypertension [37,38,39]. A systematic review and meta-analysis based on 305,432 participants from 53 studies in Bangladesh reported the pooled prevalence of hypertension at 41% [38]. There are data indicating that people are not adequately aware of several chronic diseases and their associated risk factors and complications [40,41,42]. Islam et al. [40,42] reported that 50% of the general population of a rural district did not know that physical inactivity was a risk factor for diabetes, and 58% of people did not know that diabetes-related vision loss could be prevented. None of the previous studies in Bangladesh have reported the knowledge of the health benefits of physical activity and attitudes to organize and participate in physical activity programs. This pilot study aimed to assess the knowledge of and attitudes towards participating in physical activity programs among people who reported low physical activity reported through the GPAQ-2 questionnaire [34] among adults with hypertension in a rural area in Bangladesh as an initial investigation.

## 2. Materials and Methods

### 2.1. Study Location

The present study involved baseline data collected as part of a cluster randomized controlled trial (RCT) for lowering blood pressure by changing lifestyle conducted in 307 participants aged 30–75 years in the Banshgram Union of the Narail District in Bangladesh. The study location was situated approximately 200 km away from the capital city Dhaka. Participants from the cross-sectional Bangladesh Population-based Diabetes and Eye Study who had been previously diagnosed with stage 1 hypertension [39] were the source for the current investigation. A detailed descript of the protocol and study cohort has been published previously [43]. We recruited 154 participants from cluster 1, the intervention arm, and 153 from cluster 2, the control arm. Bangladesh is divided into 64 districts; each district is divided into 3–8 Upazila, each Upzilla is partitioned into 12–18 Unions, and each Union consist of 15–20 villages. Banshgram is such a Union, with 18 villages [44].

### 2.2. Statistical Power

The sample size was determined based on statistical power to estimate positive attitudes towards physical activity. A previous study [24] reported that 26% of adults had positive attitudes towards physical activity. The sample size of 307 was adequate to detect the proportion of 26% positive attitudes towards participating in any physical activity programs with 10% of marginal error. The sample size has a statistical power of at least 90% and a significance level of 0.05.

### 2.3. Recruitment

The current study utilized baseline data collected from December 2020 to January 2021. A local NGO—the Organization for Rural Community Development (ORCD) in Bangladesh (www.orcdbd.org)—and its investigators were involved in recruitment and data collection. The local investigators and the data collectors received training from the chief investigator through four Zoom meetings to understand the study protocol and data collection. The investigators and the data collectors set up communication with the potential participants over the phone or through direct contact. Upon establishment of contact with potential participants, they were assessed for inclusion and exclusion. The inclusion criteria were: (i) clinic blood pressure more than or equal to 130/80 mm Hg who were not taking medication, (ii) blood pressure < 130/80 but using anti-hypertensive medication for a minimum of six weeks, and (iii) living in the Banshgram Union only. The exclusion criteria were: (i) aged > 75 years of age, (ii) pregnant, and (iii) advanced CVDs or who had any serious condition that restricted their participation in the study. The study location, the sources population’s demographic characteristics, and the cluster RCT have been described in detail [43,45].

### 2.4. Participants Benefits

The study involved volunteer participation. However, we provided 20 Omron blood pressure measuring units to 20 team leaders, one machine per leader, who monitored the blood pressure of 15 participants’ during the intervention [45]. After the intervention, the blood pressure devices were not taken back, allowing future blood pressure monitoring in the community.

### 2.5. Outcome Measures: Awareness of Health Benefits of and Attitudes towards Taking Part in Physical Activity

We used the Global Physical Activity Questionnaire version 2 (GPAQ-2) [34], developed by the World Health Organization (WHO) for physical activity surveillance in developing countries, to measure physical activity levels. The GPAQ-2 questionnaire included physical activity-related and sedentary behavior-related questions. Physical activity was measured based on physical activity at work, transportation (travel to and from places), and recreational activities, such as participating in any sports programs. People who spent less than 2.5 h of physical activity per week were considered to have low physical activity levels and were further asked questions related to knowledge, attitudes, and practice of physical activity. Five items were used to assess awareness of the health benefits of physical activity and attitudes towards taking part in physical activity programs. Items were adapted from a smoking cessation motivation questionnaire (Q-MAT) [46].

Awareness of health benefits and attitudes towards physical activity were assessed by asking participants

(i) Is physical activity good for health with possible answers “not at all”, “a little”, “a lot”, and “enormous”.
(ii) Are you interested in taking part in any regular physical activity programs, with possible answers “yes” or “no”;
(iii) At the moment, are you considering organizing a regular exercise program with your peers, with possible answers “yes” or “no”;
(iv) At the moment are you considering participating in a regular exercise program if it is organized, with possible answers “yes” or “no”;
(v) Do you feel unhappy if you do not do exercise, with possible answers “never”, “sometimes”, or “often”.

### 2.6. Sociodemographic Factors

The following sociodemographic factors were recorded including age, gender, and the highest level of education—categorized as no schooling, primary to high school (grade 1 to 9), secondary school certificate, or any higher-level education. Socioeconomic status was classified as poor, middle class, or rich, assessed according to Cheng et al. [47]. Occupation was categorized as a farmer, homemaker, self-managed business, laborers in professions that include digging soils, pulling a rickshaw, or any laborious works, and government and non-government employees.

### 2.7. Questionnaire Rigour and Preparation

The questionnaire was prepared first in English before being translated into Bengali separately by a local senior educator and the principal investigator. Upon agreement of two translators to use the questionnaire for data collection, the two translated versions were combined and used for pre-testing. The questionnaire was pilot-tested with ten adults with hypertension who were not included in the final study. The pilot test was performed to assess the comprehension, wording, and appropriateness of the questionnaire. The questionnaire was used previously to determine awareness and motivation to participate in a smoking cessation program in low–middle-income countries [48]. However, the instrument was not validated on its own as a tool in Bangladesh, but it was pre-tested.

### 2.8. Statistical Analysis

Proportions of participants taking part in at least 2.5 h of physical activity per week and those less than 2.5 h per week were reported by participants’ characteristics, including sex, age, level of education, and occupation using Chi-square tests. Awareness of health benefits and attitudes towards organizing or participating in physical activity for individual items by sociodemographic factors was analyzed using Chi-square tests. Rasch analysis was used to convert categorical responses to logit scores and, in turn, into an interval and linear scale to create a combined score from all the items, which provided a valid endorsement on the continuum. Researchers generally report either positive or negative attitudes based on binary outcomes “Yes” or “No” or by combining multicategories into two categories. Such methods are likely to introduce misclassification and are biased towards the positive [24,49,50]. The combined score ranged from zero to 100; zero indicates complete unawareness and having complete negative attitudes towards organizing or participating in any physical activities, 100 indicates full endorsement of all items, and above 50 indicates above-average endorsement of all items combined.

We applied a generalized linear model (GLM) to report the endorsement percentage for different factors—without adjustment for covariates and after adjustment for age, sex, level of education, socioeconomic status and occupation. The binary logistic regression technique was used to report the association of the sociodemographic variables with the outcome variable of above-average endorsement of the knowledge and attitudes items’ combined score. Statistical software SPSS (IBM SPSS Statistics for Windows, Version 27.0. IBM Corp, Armonk, NY, USA) was used for statistical analyses, and RUMM 30 [51] was used for Rasch analysis.

## 3. Results

Overall, 135 (44%) participants reported that they did not take part in physical activity for more than 2.5 h per week; this percentage was significantly higher with increasing age (24% in people aged 30–39 years, 31% in 40–49 years, 40% in 50–59 years, 62% in 60–69 years, and 77% in 70–75 years of age, *p* < 0.001 for trend), and among employees compared to farmers (72% vs. 26%). People who took part in more than 2.5 h of weekly physical activity had lower systolic blood pressure, 146 (17)/89 (9) vs. 152 (21)/91 (11) mmHg [systolic (SD)/diastolic (SD)], compared with those who did not take part in 2.5 h of weekly physical activity. Gender (45.5% women vs. 42.5% men, *p* = 0.60), level of education (51.5% no education, 40.5% primary to high school, and 39.0% school secondary certificate (SSC) or above), SES, or diabetes status was not associated with physical inactivity measured by less than 2.5 h of physical activity per week. According to metabolic equivalent task (MET)-min physical activity, 16% had less than 600 MET-min per week and 65% had a high physical activity level of more than 3000 MET-min per week. Systolic blood pressure was higher in the increased physical activity group >3000 MET-min per week than those in the low physical activity group, measured as less than 600 MET-min per week. Older people, people with low education levels, homemakers, and people with diabetes were associated with lower MET-min per week (Table 1).

Table 2 shows the perception of and intention to participate in physical activity among people who were not active for more than 2.5 h per week. A total of 131 participants (97%) agreed that physical activity was good for health, 115 (85%) participants were interested in participating in physical exercise, and half of the farmers were not interested in any physical activity. Men and younger people were more likely to attend any physical activity program if it was organized (35.4% of men vs. 15.7% of women, *p* = 0.003, and 63.6% of people of age 30–39 years, 17.2% of age 40–59 years, and 25.8% of people age 60 years or older, *p* = 0.02, were very interested in participating in any physical activity programs).

The results of the Rasch analysis showed that positive perceptions and intention to participate in physical activity was below the average of the response category continuum, mean (95% CI), endorsement was 46.6% (43.3, 49.8), which was significantly lower among women (40.6%) compared to men (53.1%, *p* < 0.001). After adjusting for age and gender, people with no education (mean (95% CI) 42.7 (36.2, 49.2)) and with low SES (mean (95% CI) 44.0 (36.8, 51.2)) were less likely to have positive perceptions and intention to take part in any physical activities compared to higher education and higher SES, respectively. In total, 33% of farmers were likely to organize or participate in any physical activity programs, i.e., one-third of the response category continuum (Table 3).

Overall, 48.8% of 135 less physically active participants had above-average positive attitudes in organizing or participating in physical activity. Compared to women men (odds ratio (OR) (95% Confidence interval (CI)) 3.50 (1.72, 7.11)), compared to people with no education people with primary to high school level of education (OR (95% CI) 3.06 (1.27, 7.38)) and secondary school certificate or above (OR (95% CI) 8.85 (2.33, 33.6)), and compared to people with low SES people with better SES (OR (95% CI) 2.53 (1.07, 6.0)) were associated with above-average endorsement of positive attitudes towards physical activity (Table 4).

## 4. Discussion

In this study, we aimed to assess the knowledge of and attitudes towards participating in physical activity programs among people with hypertension who reported that they took part in less than 2.5 h of physical activity per week. To improve participation in physical activity, it is essential to know about the health benefits of physical activity and attitudes towards organizing and taking part in any physical activities. Understanding the benefits of physical activity may be expected to change people’s attitudes towards physical activity. Key findings from this study were: (1) in total, 44% of people indicated that they took part in less than 2.5 h of physical activity per week. According to the WHO recommended cut point of less than 600 MET-min per week, only 16% of people were physically inactive. The PA level was reported to decrease as people become older. (2) In total, 95% of people were aware of the health benefits of physical activity, and the majority of the participants were interested in participating in organized physical activity programs. (3) Only 10% of participants were interested in organizing any physical activity programs. (4) Peoples’ perception and attitudes towards participating and organizing physical activity were below average on the knowledge and attitudes items continuum. (5) Men, individuals with at least a primary level of education, or those from higher SES were associated with positive attitudes towards physical activity (above-average score on the continuum).

According to the WHO recommendation of physical activity measured by MET-min per week [52], our study indicated that a higher proportion of people were physically active. Still, many people responded that they participated in less than 2.5 h of physical activity per week, and the PA levels drop as people become older. Our findings are consistent with previous studies in Bangladesh [30,31,32,33] and other low–middle-income countries [27,28,29].

Our study showed that attitudes towards physical activity are comparable with previous studies [23,53,54,55]. Saleh et al. [53] studied the knowledge, attitudes, and practice of physical exercise to reduce body weight to control blood glucose in patients with diabetes in a hospital-based sample. The study reported that most people were aware of the health benefits of physical activity and would participate in any physical activity programs to manage weight. Our study demonstrates that people have sufficient knowledge about the health benefits of physical activity. However, knowledge alone is not sufficient for individuals to initiate and sustain physical activity programs; positive attitudes and motivation to change practices are reported to be predictors of physical activity [55,56]. Positive attitudes towards participation in physical activity programs have been shown to be important [57].

The majority of the participants had positive attitudes towards participating in any exercise programs if it was organized and was consistent with previous studies with positive attitudes towards participation in physical activity [49,53,55]. Rahman et al. [55] studied the effects of attitude, motivation, and eagerness for physical activity among middle-aged and older Chinese adults and reported that about 90% of people were interested in attending any physical activity program. Another study of 200 individuals with hypertension in Nigeria [49] reported that 80% of participants believed that they would participate in a physical activity program in order to manage hypertension based on a single-item response. In our study, fewer than half of the participants had positive attitudes towards physical activity measured on the response category continuum on a linear scale based on all items. Thus, our results are not directly comparable. In our study, the combined attitude score was greatly influenced by people’s inner feelings. A small proportion of people felt unhappy if they were not participating in any physical activity program. Another item—“consider organizing physical activity program”—contributed to the middle range of the attitude score continuum. This indicates that people are generally interested in attending physical activity programs but are reluctant to organize such programs and do not feel obligated to participate in such programs. Positive intention to participate by organizing physical activity programs can predict sustainable involvement and participation in physical activity [55,58]. The current study found that men, people with higher education levels, those from higher SES, and employees or businesspersons were more likely to have positive attitudes towards organizing or participating in any physical activities. Association of higher educational level and higher socio-economic status with higher awareness or positive attitudes towards physical activity in managing chronic diseases including hypertension has previously been shown [40,42,49,59].

The current study has significant implications. Due to the increase in the prevalence of hypertension, it is essential to increase health awareness and positive attitudes towards organizing and participating in physical activity programs to control blood pressure. In the current study, the Rasch analysis converted the categorical scale into a continuous linear scale. Thus, it provided a valid endorsement on the continuum by addressing the possible bias towards the positive or negative that usually raises when binary outcomes are used, i.e., “Yes” or “No”, or when multicategories are combined into two categories to report positive or negative attitudes [24,49,50]. Therefore, the present study provides further insight into the attitudes towards organizing or participating in physical activity programs and their associated factors. The strengths of the study include the face-to-face data collection methods. The study also involved almost 50% of women’s participation. However, the study was not free from all limitations; for example, data collection was from a single rural location, which restricts generalization at the national level. Another limitation was the use of the Q-MAT questionnaire, which was not specifically developed to measure knowledge of and attitudes toward physical activity.

## 5. Conclusions

People are aware of the health benefits of physical activity and have positive attitudes towards participating in any physical activity. However, people are reluctant to organize and do not feel obligated to participate in physical activity programs. Men, people with higher education levels, and people with higher SES were associated with above-average endorsement of the physical activity programs. Health promotion programs need to be organized for increasing motivation to organize and participate in physical activity programs. Personalized feedback about physical activity may be an essential first step to attitude and behavior change.

## Figures and Tables

**Table 1:** Characteristics of the participants according to their reported physical activity.

		≥2.5 or <2.5 h Activities per Week			Total Activity, Metabolic Equivalent Task (MET)-Min per Day			
	Number	≥2.5 h	<2.5 h	*p*	≥3000 MET-Min	600–2999 MET-Min	<600 MET-Min	*p*
Factors		*n* (%)	*n* (%)		*n* (%)	*n* (%)	*n* (%)	
Total	307	172 (56.0)	135 (44)		199 (64.8)	58 (18.9)	50 (16.3)	
Blood pressure levels								
Systolic BP, mean (SD)		146 (16.5)	152 (20.5)	0.008	147 (17.2)	149 (16.7)	157 (23.4)	0.003
Diastolic BP, mean (SD)		89 (8.6)	91 (10.8)	0.05	89.0 (8.9)	90.4 (10)	92.5 (12)	0.07
Gender								0.19
Female	154	84 (54.5)	70 (45.5)	0.60	95(61.7)	28(18.2)	31(20.1)	
Male	153	88 (57.5)	65 (42.5)		104(68)	30(19.6)	19(12.4)	
Age, years								<0.001
Less than 40	46	35 (76.1)	11 (23.9)	<0.001	36 (78.3)	10 (21.7)	0 (0)	
40–49	65	45 (69.2)	20 (30.8)		45 (69.2)	14 (21.5)	6 (9.2)	
50–59	95	57 (60.0)	38 (40.0)		72 (75.8)	9 (9.5)	14 (14.7)	
60–69	79	30 (38.0)	49 (62.0)		37 (46.8)	20 (25.3)	22 (27.8)	
70–75	22	5 (22.7)	17 (77.3)		9 (40.9)	5 (22.7)	8 (36.4)	
Education levels								<0.001
No education	99	48 (48.5)	51 (51.5)	0.17	53 (53.5)	17 (17.2)	29 (29.3)	
Primary to high school	148	88 (59.5)	60 (40.5)		105 (70.9)	28 (18.9)	15 (10.1)	
SSC or above	59	36 (61.0)	23 (39)		41 (69.5)	13 (22)	5 (8.5)	
SES								0.81
Poor	92	56 (60.9)	36 (39.1)	0.25	62 (67.4)	16 (17.4)	14 (15.2)	
Middle class or rich	214	115 (53.7)	99 (46.3)		136 (63.6)	42 (19.6)	36 (16.8)	
Occupation								0.001
Farmer	66	49 (74.2)	17 (25.8)	<0.001	57 (79.2)	8 (11.1)	7 (9.7)	
Homemakers	146	84 (57.5)	62 (42.5)		93 (63.7)	27 (18.5)	26 (17.8)	
Employees	53	15 (28.3)	38 (71.7)		26 (49.1)	12 (22.6)	15 (28.3)	
Businesspersons	24	13 (54.2)	11 (45.8)		15 (62.5)	9 (37.5)	0 (0)	
Diabetes status								0.002
No diabetes	217	118 (54.4)	99 (45.6)	0.10	137 (63.1)	36 (16.6)	44 (20.3)	
Diabetes	41	20 (48.8)	21 (51.2)		22 (53.7)	14 (34.1)	5 (12.2)	
Unknown	49	34 (69.4)	15 (30.6)		40 (81.6)	8 (16.3)	1 (2)	

**Table 2:** Perception of physical activity (PA) and intention to take part in physical activity programs among 135 people who reported to be physically less active.

		Total	Gender			Age Group, Years				Occupation				
			Female	Male		<40	40–59	60+		Farmer	Housewife	Service	Business	
		*n*(%)	*n* (%)	*n* (%)	*p*	*n* (%)	*n* (%)	*n* (%)	*p*	*n* (%)	*n* (%)	*n* (%)	*n* (%)	*p*
Is PA good for health?	not at all	4 (3)	4 (5.7)	0 (0)	0.002	0 (0)	3 (5.2)	1 (1.5)	0.61	0 (0)	4 (6.5)	0 (0)	0 (0)	0.07
	a little	67 (49.6)	41 (58.6)	26 (40)		4 (36.4)	30 (51.7)	33 (50)		6 (35.3)	37 (59.7)	16 (42.1)	4 (36.4)	
	a lot	64 (47.4)	25 (35.7)	39 (60)		7 (63.6)	25 (43.1)	32 (48.5)		11 (64.7)	21 (33.9)	22 (57.9)	7 (63.6)	
Are you Interested in PA	No	20 (14.8)	9 (12.9)	11 (16.9)	0.63	0 (0)	10 (17.2)	10 (15.2)	0.47	9 (52.9)	8 (12.9)	3 (7.9)	0 (0)	<0.001
	Yes	115 (85.2)	61 (87.1)	54 (83.1)		11 (100)	48 (82.8)	56 (84.8)		8 (47.1)	54 (87.1)	35 (92.1)	11 (100)	
Consider organising exercise program	not at all	14 (10.4)	8 (11.4)	6 (9.2)	0.05	1 (9.1)	7 (12.1)	6 (9.1)	0.80	0 (0)	7 (11.3)	5 (13.2)	2 (18.2)	0.14
	a little	90 (66.7)	52 (74.3)	38 (58.5)		6 (54.5)	38 (65.5)	46 (69.7)		13 (76.5)	45 (72.6)	23 (60.5)	4 (36.4)	
	a lot	31 (23)	10 (14.3)	21 (32.3)		4 (36.4)	13 (22.4)	14 (21.2)		4 (23.5)	10 (16.1)	10 (26.3)	5 (45.5)	
Attend if any program is organised	not at all	3 (2.2)	3 (4.3)	0 (0)	0.003	0 (0)	2 (3.4)	1 (1.5)	0.02	0 (0)	3 (4.8)	0 (0)	0 (0)	0.45
	A little	98 (72.6)	56 (80)	42 (64.6)		4 (36.4)	46 (79.3)	48 (72.7)		12 (70.6)	48 (77.4)	26 (68.4)	7 (63.6)	
	a lot	34 (25.2)	11 (15.7)	23 (35.4)		7 (63.6)	10 (17.2)	17 (25.8)		5 (29.4)	11 (17.7)	12 (31.6)	4 (36.4)	
Do you feel unhappy if you don’t do exercise?	never	42 (31.1)	27 (38.6)	15 (23.1)	0.17	5 (45.5)	19 (32.8)	18 (27.3)	0.25	7 (41.2)	23 (37.1)	8 (21.1)	2 (18.2)	0.43
	sometime	84 (62.2)	38 (54.3)	46 (70.8)		4 (36.4)	36 (62.1)	44 (66.7)		10 (58.8)	34 (54.8)	27 (71.1)	8 (72.7)	
	Often	9 (6.7)	5 (7.1)	4 (6.2)		2 (18.2)	3 (5.2)	4 (6.1)		0 (0)	5 (8.1)	3 (7.9)	1 (9.1)	

**Table 3:** Endorsement in positive perception and intention to take part in physical activity among 135 people who were less active, and their associated factors.

Characteristics	No of Participants	Percentage of Endorse (95% CI) *	*p*	Percentage of Endorse (95% CI)	*p*
Total	135	46.6 (43.3, 49.9)			
Sex			<0.001		
Female	70	40.6 (36.3, 44.9)			
Male	65	53.1 (48.2, 57.9)			
Age, years					
Below 40	11	57.7 (47.0, 68.5)	0.06		
40–49	20	46.7 (36.8, 56.5)			
50–59	38	41.2 (35.2, 47.1)			
60–69	49	45.7 (40.6, 50.9)			
70–75	17	54.0 (41.3, 66.7)			
Level of education					
No education	51	38.4 (33.8, 43)	<0.001	42.7 (36.2, 49.2)	0.006
Primary to high school	60	47.7 (42.8, 52.5)		49.7 (44.7, 54.8)	
SSC or above	23	59.6 (51.7, 67.6)		58.6 (50.7, 66.5)	
SES					
Poor	36	39.6 (33, 46.1)	0.01	44 (36.8, 51.2)	0.04
Middle class	99	49.2 (45.3, 53)		51.4 (47, 55.9)	
Occupation					
Farmer	19	43.3 (34.8, 51.8)	0.005	36.7 (24.8, 48.5)	0.08
Homemakers	62	41 (36.3, 45.7)		53.1 (44.2, 61.9)	
Employees	38	53.3 (46.8, 59.9)		50.4 (42.3, 58.6)	
Businesspersons	11	55.5 (43.7, 67.4)		47.2 (34, 60.4)	
Diabetes status					
No diabetes	99	44.8 (41.1, 48.6)	0.21		
Diabetes	21	52.1 (40.9, 63.3)			
Unknown	15	50.7 (41.3, 60)			

* Percentage of endorsement mean (95% confidence interval (CI)) (unadjusted); † mean (95% CI) adjusted for age, gender, education (except for education), and SES (except for SES).

**Table 4:** Endorsement of positive perception and intention to take part in physical activity among 136 people who were less active, and their associated factors.

Characteristics	No of Participants	Attitudes towards Positive	OR (95% CI)	OR (95% CI) †
Total	135	66 (48.8%)		
Sex				
Female	70	24 (34.3)	1.00 (reference)	
Male	65	42 (64.6)	3.50 (1.72, 7.11)	
Age, years				
Below 40	11	8 (72.7)	1.00	
40–49	20	9 (45.0)	0.31 (0.06, 1.51)	
50–59	38	15 (39.5)	0.25 (0.06, 1.07)	
60–69	49	24 (49.0)	0.36 (0.09, 1.52)	
70–75	17	10 (58.8)	0.54 (0.10, 2.77)	
Level of education				
No education	51	13 (25.5)	1.00 (reference)	1.00 (reference)
Primary to high school	60	33 (55.0)	3.57 (1.59, 8.03)	3.06 (1.27, 7.38)
SSC or above	23	19 (82.6)	13.9 (3.98, 48.4)	8.85 (2.33, 33.6)
SES				
Poor	36	11 (30.6)	1.00 (reference)	1.00 (reference)
Middle class	99	55 (55.6)	2.84 (1.26, 6.40)	2.53 (1.07, 6.0)
Occupation				
Farmer	19	9 (47.4)	1.00 (reference)	
Housewife	62	62 (35.5)	0.61 (0.22, 1.73)	
Service holder	38	38 (63.2)	1.91 (0.62, 5.81)	
Business	11	9 (81.8)	5.00 (0.85, 29.6)	
Diabetes status				
No diabetes	99	45 (45.5)	1.00 (reference)	
Diabetes	21	13 (61.9)	1.95 (0.73, 5.12)	
Unknown	15	8 (53.3)	1.37 (0.46, 4.07)	

† OR (95% CI) adjusted for age, gender, education (except for education), and SES (except for SES).

## Data Availability

Data will be made available on reasonable request from the corresponding author.

## References

[1] WHO (2019). Hypertension Fact Sheet. https://www.who.int/news-room/fact-sheets/detail/hypertension.

[2] Institute for Health Metrics and Evaluation (IHME) (2019). High Systolic Blood Pressure-Level 2 Risk.

[3] Zhao R., Bu W., Chen Y., Chen X. (2020). The Dose-Response Associations of Sedentary Time with Chronic Diseases and the Risk for All-Cause Mortality Affected by Different Health Status: A Systematic Review and Meta-Analysis. J. Nutr. Health Aging.

[4] Nocon M., Hiemann T., Muller-Riemenschneider F., Thalau F., Roll S., Willich S.N. (2008). Association of physical activity with all-cause and cardiovascular mortality: A systematic review and meta-analysis. Eur. J. Cardiovasc. Prev. Rehabil..

[5] Katzmarzyk P.T., Lear S.A. (2011). Physical activity for obese individuals: A systematic review of effects on chronic disease risk factors. Obes. Rev..

[6] Owen N., Healy G.N., Dempsey P.C., Salmon J., Timperio A., Clark B.K., Goode A.D., Koorts H., Ridgers N.D., Hadgraft N.T. (2020). Sedentary Behavior and Public Health: Integrating the Evidence and Identifying Potential Solutions. Annu. Rev. Public Health.

[7] Climie R., Srikanth V., Keith L.J., Davies J.E., Sharman J.E. (2015). Exercise excess pressure and exercise-induced albuminuria in patients with type 2 diabetes mellitus. Am. J. Physiol. Circ. Physiol..

[8] Lee I.-M., Shiroma E.J., Lobelo F., Puska P., Blair S.N., Katzmarzyk P.T. (2012). Lancet Physical Activity Series Working Group. Effect of physical inactivity on major non-communicable diseases worldwide: An analysis of burden of disease and life expectancy. Lancet.

[9] Warburton D.E., Bredin S.S. (2017). Health benefits of physical activity. Curr. Opin. Cardiol..

[10] U.S. Department of Health and Human Services (2018). Physical Activity Guidelines for Americans.

[11] WHO (2013). Global Action Plan for the Prevention and Control of NCDs 2013–2020. https://www.who.int/publications/i/item/9789241506236.

[12] Lear S.A., Hu W., Rangarajan S., Gasevic D., Leong D., Iqbal R., Casanova A., Swaminathan S., Anjana R.M., Kumar R. (2017). The effect of physical activity on mortality and cardiovascular disease in 130 000 people from 17 high-income, middle-income, and low-income countries: The PURE study. Lancet.

[13] Carey R.M., Muntner P., Bosworth H.B., Whelton P.K. (2018). Prevention and Control of Hypertension. J. Am. Coll. Cardiol..

[14] Dorans K.S., Mills K.T., Liu Y., He J. (2018). Trends in Prevalence and Control of Hypertension According to the 2017 American College of Cardiology/American Heart Association (ACC/AHA) Guideline. J. Am. Hear. Assoc..

[15] De Souza A.C.C., Borges J.W.P., Moreira T.M.M. (2016). Quality of life and treatment adherence in hypertensive patients: Systematic review with meta-analysis. Revista de Saúde Pública.

[16] Müller-Riemenschneider F., Reinhold T., Nocon M., Willich S.N. (2008). Long-term effectiveness of interventions promoting physical activity: A systematic review. Prev. Med..

[17] Orrow G., Kinmonth A.-L., Sanderson S., Sutton S. (2012). Effectiveness of physical activity promotion based in primary care: Systematic review and meta-analysis of randomised controlled trials. BMJ.

[18] Bauman A.E., Reis R.S., Sallis J.F., Wells J.C., Loos R.J.F., Martin B.W. (2012). Correlates of physical activity: Why are some people physically active and others not?. Lancet.

[19] Graham G. (1995). Physical education through students’ eyes and in students’ voices: Introduction. J. Teach. Phys. Educ..

[20] Sarafino E.P. (2011). Health Psychology: Biopsychosocial Interactions.

[21] Vega W.A., Sallis J.F., Patterson T., Joan R., Atkins C., Nader P.R. (1987). Assessing knowledge of cardiovascular health-related diet and exercise behaviors in anglo- and Mexican-Americans. Prev. Med..

[22] Plotnikoff R.C., Brunet S., Courneya K.S., Spence J.C., Birkett N.J., Marcus B.H., Whiteley A.J. (2007). The Efficacy of Stage-Matched and Standard Public Health Materials for Promoting Physical Activity in the Workplace: The Physical Activity Workplace Study (PAWS). Am. J. Heal. Promot..

[23] Veluswamy S.K., Maiya A.G., Nair S., Guddattu V., Nair N.S., Vidyasagar S. (2014). Awareness of chronic disease related health benefits of physical activity among residents of a rural South Indian region: A cross-sectional study. Int. J. Behav. Nutr. Phys. Act..

[24] Awotidebe T.O., Adedoyin R.A., Rasaq W.A., Adeyeye V.O., Mbada C.E., Akinola O.T., Otwombe K.N. (2014). Knowledge, Atti-tude and Practice of Exercise for Blood Pressure Control: A Cross-sectional Survey. J. Exerc. Sci. Phys.-Ther..

[25] Troiano R.P., Berrigan D., Dodd K.W., Mâsse L.C., Tilert T., Mcdowell M. (2008). Physical Activity in the United States Measured by Accelerometer. Med. Sci. Sports Exerc..

[26] Godino J.G., Watkinson C., Corder K., Sutton S., Griffin S.J., van Sluijs E.M. (2014). Awareness of physical activity in healthy middle-aged adults: A cross-sectional study of associations with sociodemographic, biological, behavioural, and psychological factors. BMC Public Health.

[27] Pedisic Z., Shrestha N., Loprinzi P.D., Mehata S., Mishra S.R. (2019). Prevalence, patterns, and correlates of physical activity in Nepal: Findings from a nationally representative study using the Global Physical Activity Questionnaire (GPAQ). BMC Public Health.

[28] Guwatudde D., Kirunda B.E., Wesonga R., Mutungi G., Kajjura R., Kasule H., Muwonge J., Bahendeka S.K. (2016). Physical Activity Levels Among Adults in Uganda: Findings From a Countrywide Cross-Sectional Survey. J. Phys. Act. Health.

[29] Padrão P., Damasceno A., Silva-Matos C., Prista A., Lunet N. (2012). Physical activity patterns in Mozambique: Urban/rural differences during epidemiological transition. Prev. Med..

[30] Moniruzzaman M., Zaman M.M., Islalm M., Ahasan H., Kabir H., Yasmin R. (2016). Physical activity levels in Bangladeshi adults: Results from STEPS survey 2010. Public Health.

[31] Zaman M.M., Rahman M., Rahman R., Bhuiyan M.R., Karim N., Chowdhury A.J. (2016). Prevalence of risk factors for non-communicable diseases in Bangladesh: Results from STEPS survey 2010. Indian J. Public Health.

[32] Moniruzzaman M., Ahmed M.S.A.M., Zaman M.M. (2017). Physical activity levels and associated socio-demographic factors in Bangladeshi adults: A cross-sectional study. BMC Public Health.

[33] Islam F. (2021). Factors Associated with Physical Activity among People with Hypertension in a Rural Area in Bangladesh: Baseline Data from a Cluster Randomized Control Trial. Int. J. Environ. Res. Public Health.

[34] WHO GPAQ: Global Physical Activity Questionnaire (Version 2.0). Department of Chronic Diseases and Health Promotionl.

[35] Conner M., Sparks P., Conner M., Norman P. (2005). The theory of planned behavior and health behaviors. Predicting Health Behavior.

[36] Busari O.A., Olanrewaju T.O., Desalu O.O., Opadijo O.J., Jimoh A.K., Agboola S.M., Busari O.E., Olalekan O. (2010). Impact of Patients’ Knowledge, Attitude and Practices on Hypertension on Compliance with Antihypertensive Drugs in a Resource-poor Setting. TAF Prev. Med. Bull..

[37] Saquib N., Saquib J., Ahmed T., Khanam M.A., Cullen M.R. (2012). Cardiovascular diseases and Type 2 Diabetes in Bangladesh: A systematic review and meta-analysis of studies between 1995 and 2010. BMC Public Health.

[38] Chowdhury M.Z.I., Rahman M., Akter T., Akhter T., Ahmed A., Shovon M.A., Farhana Z., Chowdhury N., Turin T.C. (2020). Hypertension prevalence and its trend in Bangladesh: Evidence from a systematic review and meta-analysis. Clin. Hypertens..

[39] Islam F.M.A., Bhuiyan A., Chakrabarti R., Rahman M.A., Kanagasingam Y., Hiller J. (2015). Undiagnosed hypertension in a rural district in Bangladesh: The Bangladesh Population-based Diabetes and Eye Study (BPDES). J. Hum. Hypertens..

[40] Islam F.M.A., Chakrabarti R., Islam S.Z., Finger R.P., Critchley C. (2015). Factors Associated with Awareness, Attitudes and Practices Regarding Common Eye Diseases in the General Population in a Rural District in Bangladesh: The Bangladesh Population-based Diabetes and Eye Study (BPDES). PLoS ONE.

[41] Uddin M.N., Bhar S., Islam F.M.A. (2019). An assessment of awareness of mental health conditions and its association with socio-demographic characteristics: A cross-sectional study in a rural district in Bangladesh. BMC Heal. Serv. Res..

[42] Islam F.M.A., Chakrabarti R., Dirani M., Islam M.T., Ormsby G., Wahab M., Critchley C., Finger R.P. (2014). Knowledge, Attitudes and Practice of Diabetes in Rural Bangladesh: The Bangladesh Population Based Diabetes and Eye Study (BPDES). PLoS ONE.

[43] Islam F.M.A., Lambert E.A., Islam S.M.S., Islam M.A., Biswas D., McDonald R., Maddison R., Thompson B., Lambert G.W. (2021). Lowering blood pressure by changing lifestyle through a motivational education program: A cluster randomized controlled trial study protocol. Trials.

[44] Bangladesh Bureau of Statistics (2011). Population and Housing Census. http://www.bbs.gov.bd/site/page/47856ad0-7e1c-4aab-bd78-892733bc06eb/Population-and-Housing-Census.

[45] Chakrabarti R., Finger R.P., Lamoureux E., Islam M.T., Dirani M., Bhuiyan M.A. (2015). Rationale and methodology for a popula-tion-based study of diabetes and common eye diseases in a rural area in Bangladesh: Bangladesh Population-based Diabetes and Eye Study (BPDES). Bangladesh J. Med. Sci..

[46] Aubin H., Lagrue G., Legeron P., Pelissolo G.A. (2004). Smoking Cessation Motivation Questionnaire (Q-MAT): Construction and Validation.

[47] Cheng Y., Chi I., Boey K., Ko L., Chou K. (2002). Self-rated economic condition and the health of elderly persons in Hong Kong. Soc. Sci. Med..

[48] Hessami Z., Sharifi H., Heydari G., Masjedi M. (2012). Is motivational Q-mat test useful to predict smoking cessation?. Eur. Respir. J..

[49] Maruf F.A., Ojukwu C.C., Akindele M.O. (2018). Perception, Knowledge, and Attitude toward Physical Activity Behaviour: Implications for Participation among Individuals with Essential Hypertension. High. Blood Press. Cardiovasc. Prev..

[50] Awotidebe T.O., Adedoyin R.A., Afolabi M.A., Opiyo R. (2016). Knowledge, attitude and practice of exercise for plasma blood glucose control among patients with type-2 diabetes. Diabetes Metab. Syndr. Clin. Res. Rev..

[51] RUMM (2010). For Analysing Assessment and Attitude Questionnaire Data. http://www.rummlab.com.au/.

[52] World Health Organization (2010). Global Recommendations on Physical Activity for Health.

[53] Saleh F., Mumu S.J., Ara F., Ali L., Hossain S., Ahmed K.R. (2012). Knowledge, attitude and practice of type 2 diabetic patients regarding obesity: Study in a tertiary care hospital in Bangladesh. J. Public Health Afr..

[54] Harrison A.L., Taylor N.F., Shields N., Frawley H.C. (2018). Attitudes, barriers and enablers to physical activity in pregnant women: A systematic review. J. Physiother..

[55] Rahman M., Gu D., Liang C., Rashid R.M., Akter M. (2020). Effects of Attitude, Motivation, and Eagerness for Physical Activity among Middle-Aged and Older Adults. J. Health Eng..

[56] Biddle S., Mutrie N. (2001). Psychology of Physical Activity: Deter-Minants, Well-Being and Interventions.

[57] Rikard G.L., Banville D. (2006). High school student attitudes about physical education. Sport Educ. Soc..

[58] Mikalsen H.K., Lagestad P., Bentzen M., Säfvenbom R. (2019). Does Eagerness for Physical Activity Matter? The Association Between Eagerness and Physical Activity Among Adolescents. Front. Public Health.

[59] Islam F.M.A., Kawasaki R., Finger R.P. (2018). Factors associated with participation in a diabetic retinopathy screening program in a rural district in Bangladesh. Diabetes Res. Clin. Pract..

